# The Secretary's Statements

**Published:** 1919-09-20

**Authors:** Arthur Watts

**Affiliations:** Secretary. Queen Charlotte's Lying-In Hospital


*To the Editor of* THE HOSPITAL.

### The Secretary's Statements.

*To the Editor of* THE HOSPITAL.

SIR,—With reference to the statements made by your correspondent as to the hours of duty of pupils at this hospital, I am directed by the House Committee to state that this matter has been under consideration recently, and it is hoped to be able to arrange for some diminution. The hours on duty are not so long as stated by your correspondent. The hours for day nurses are 7 A.M. to 9 P.M., with two hours off duty each day, and, of course, meal-times, which total 1¾ hours. Pupils get two (separate) days off duty each month. The chief difficulty in the way of a reduction of the hours of duty is that of increased accommodation for the consequent additional staff, and which we had hoped to provide in connection with the extension of the hospital which was about to be undertaken when war broke out, and had to be postponed. The Committee are anxious to proceed with this enlargement, and they are appealing for £40,000 for the purpose; they will then have additional accommodation for nurses which will make it possible to reduce the hours of duty for the pupils.

In considering the question of hours of duty in a lying-in hospital it is important to bear in mind the fact that nearly all the pupils are preparing for the Central Midwives Board Examination, and that for this purpose they must each personally deliver twenty cases and nurse twenty patients during their period of training. If the hours of duty were materially shortened it would mean that the

**The Editor's Letter-Box—(*continued*).**

period of training would have to be extended. Another difficulty which is perhaps not fully appreciated is the considerable fluctuation in the number of cases in the hospital from time to time.

The amount of cleaning in the wards is only what is customary elsewhere. Nurses have to cleanse the bowls and other ward utensils used for their patients, and when their patients are discharged the ward is turned out and cleaned, the floor, grate and hearth being done by the wardmaid. The nurse has to clean and boil up all ward utensils, wash and carbolise the furniture, and wash the dado of the ward. The walls and ceilings of the lying-in wards are washed down every three months, and of the labour wards every month by a man employed for this purpose. An ample supply of cleaning materials is provided, and there is no necessity for any nurse to purchase such materials. The amount of brass work is very small—in the lying-in wards there are no brass taps.

Special arrangements are made for laundry work for pupils, and the average amount paid by them is 2s. 6d. per week only, which covers all necessary laundry, and is certainly not excessive in these days of high prices.

With regard to food, every institution has experienced difficulties, both during the war and since; but the supply has been ample, and has for some time past been almost as varied as before the war. There is no need for nurses to go elsewhere for additional food, but it is not unusual for nurses of any hospital to take a meal out with friends for the sake of the greater variety obtainable at a restaurant.—Yours faithfully,

(Signed) Arthur Watts, Secretary.

Queen Charlotte's Lying-in Hospital, *September* 10, 1919.

